# Evaluation of the Expression of CCR5 and CX3CR1 Receptors and Correlation with the Functionality of T Cells in Women infected with ZIKV during Pregnancy

**DOI:** 10.3390/v13020191

**Published:** 2021-01-28

**Authors:** Débora Familiar-Macedo, Iury Amancio Paiva, Jessica Badolato-Corrêa da Silva, Fabiana Rabe de Carvalho, Helver Gonçalves Dias, Alex Pauvolid-Corrêa, Caroline Fernandes dos Santos, Mariana Gandini, Andréa Alice Silva, Silvia Maria Baeta Cavalcanti, Solange Artimos de Oliveira, Renata Artimos de Oliveira Vianna, Elzinandes Leal de Azeredo, Alba Grifoni, Alessandro Sette, Daniela Weiskopf, Claudete Aparecida Araújo Cardoso, Luzia Maria de-Oliveira-Pinto

**Affiliations:** 1 Laboratory of Viral Immunology, Fundação Oswaldo Cruz, Rio de Janeiro 1040-900, Brazil; deborafamiliar@gmail.com (D.F.-M.); iury.iap@gmail.com (I.A.P.); jessicabadolato04@gmail.com (J.B.-C.d.S.); helvergd@gmail.com (H.G.D.); carol.uned@gmail.com (C.F.d.S.); elzinandes@ioc.fiocruz.br (E.L.d.A.); 2 Multiuser Laboratory for Research in Nephrology and Medical Science, School of Medicine, Universidade Federal Fluminense, Niterói, Rio de Janeiro 24 033-900, Brazil; fabiana.r.carvalho@hotmail.com (F.R.d.C.); aasilva@id.uff.br (A.A.S.); claudetecardoso@id.uff.br (C.A.A.C.); 3 Department of Veterinary Integrative Biosciences, Texas A&M University, Texas TX, 77843, USA; pauvolid@gmail.com; 4 Laboratory of Respiratory Viruses and Measles, SARS-CoV-2 National Reference Laboratory and Regional Reference Laboratory in Americas (PAHO/WHO), Fiocruz, Rio de Janeiro 21040-900, Brazil; 5 Laboratory of Cellular Microbiology, Fundação Oswaldo Cruz, Rio de Janeiro 21040-900, Brazil; mariana.gandini@gmail.com; 6 Laboratory of Virological Diagnosis, Biomedical Institute, Universidade Federal Fluminense, Niterói, Rio de Janeiro 24 110-130, Brazil; silviacavalcanti@vm.uff.br; 7 Department of Maternal and Child, School of Medicine, Universidade Federal Fluminense, Niterói, Rio de Janeiro 24 033-900, Brazil; sartimos@id.uff.br (S.A.d.O.); renatavianna03@gmail.com (R.A.d.O.V.); 8 Center for Infectious Disease and Vaccine Research, La Jolla Institute for Immunology (LJI), La Jolla, CA 92037, USA; agrifoni@lji.org (A.G.); alex@lji.org (A.S.); dweiskopf@lji.org (D.W.); 9 Department of Medicine, Division of Infectious Diseases and Global Public Health, University of California, San Diego (UCSD), La Jolla, CA 92037, USA

**Keywords:** ZIKV, pregnancy, T cells, inflammatory response, cytotoxic activity, chemokine receptors

## Abstract

There have been reports of neurological abnormalities associated with the Zika virus (ZIKV), such as congenital Zika syndrome (CZS) in children born to mothers infected during pregnancy. We investigated how the immune response to ZIKV during pregnancy is primed and conduct a thorough evaluation of the inflammatory and cytotoxic profiles as well as the expression of CCR5 and CX3CR1. We compared the reactivity of T cells to ZIKV peptides in convalescent mothers infected during pregnancy. The child’s clinical outcome (i.e., born with or without CZS) was taken to be the variable. The cells were stimulated in vitro with ZIKV peptides and evaluated using the ELISPOT and flow cytometry assays. After in vitro stimulation with ZIKV peptides, we observed a tendency toward a higher Interferon gamma (IFN-γ)-producing T cell responses in mothers who had asymptomatic children and a higher CD107a expression in T cells in mothers who had children with CZS. We found a higher frequency of T cells expressing CD107a+ and co-expressing CX3CR1+CCR5+, which is much clearer in the T cells of mothers who had CZS children. We suggest that this differential profile influenced the clinical outcome of babies. These data need to be further investigated, including the evaluation of other ZIKV peptides and markers and functional assays.

## 1. Introduction

Reports of the incidence of microcephaly in Brazil overlapped geographically with reports of Zika virus (ZIKV) infection in 2015–2016. Oliveira Melo et al. confirmed the association between ZIKV infection and microcephaly by showing virus in amniotic fluids from two symptomatic patients. Then, this was confirmed through other studies that demonstrated that ZIKV can cross the placental barrier [1,2]. However, congenital Zika syndrome (CZS) occurs in 1 to 40% of pregnant women infected with ZIKV, which clearly shows that ZIKV infection in pregnancy is not a determinant for occurrence of CZS in the fetus [3,4,5,6].

Caires-Júnior et al. analyzed neural progenitor cells (NPCs) from three pairs of dizygotic twins who differed regarding the development of CZS. NPCs were infected in vitro with a Brazilian strain of ZIKV named ZIKVBR. Interestingly, the NPCs from children affected with CZS were more permissive to in vitro infection than were the NPCs from children not affected by CZS. Analyses on the gene expression signature of the children’s NPCs indicated significant differences in the regulatory regions of epigenetic responses. According to these authors [7], this would partly explain the greater susceptibility to ZIKV among babies with CZS than among unaffected ones. Recently, Amaral et al. compared the trophoblasts of dizygotic twins affected and not affected by CZS, by means of RNA-Seq. Similarly, trophoblasts from twins with CZS were significantly more susceptible to in vitro ZIKVBR infection than were those of unaffected children. Infection by ZIKV in the trophoblasts of twins with CZS led to negative regulation of genes relating to the organization of the extracellular matrix and to activation of leukocytes. Trophoblasts from unaffected twins secreted an increased amount of CCL5 and CXCL10 chemokines after in vitro infection. The authors concluded that trophoblasts from unaffected twins have the ability to more efficiently activate genes that play important roles in cell adhesion and in triggering the immune response to ZIKV infection in the placenta; this may contribute to predicting protection against the spreading of ZIKV in fetal tissues [8].

In addition to individual genetic factors, occurrences of congenital malformations associated with ZIKV depend on a variety of factors, including viral load and the host’s immune response.

In conjunction with the new scenario of ZIKV outbreaks around the world, mainly in regions endemic for dengue virus (DENV), efforts have been made to try to understand whether preexisting immunity directed to DENV epitopes would be able to modulate the responses of T lymphocytes to ZIKV [9,10,11,12]. Grifoni et al. contributed greatly to this regard. The latter authors demonstrated that memory T lymphocytes from individuals previously infected with DENV or immunized with the attenuated dengue tetravalent vaccine (TDLAV) recognize peptides derived from ZIKV. In addition, in patients with immunity to DENV who become infected by ZIKV, the responses of T lymphocytes to ZIKV are detected earlier and to a greater extent in the acute phase but decrease in the convalescence phase. On the other hand, in patients without immunity to DENV who become infected by ZIKV, the frequency of responses of T lymphocytes to ZIKV continues to increase in the convalescence phase, thus indicating more efficient control of viral replication and/or the elimination of ZIKV antigens in individuals with immunity to DENV. The quality of responses is also influenced by prior exposure to DENV. ZIKV-specific CD8+ T lymphocytes from patients with immunity to DENV increased the expression of granzyme B and PD-1, unlike in those without immunity to DENV [13].

Here, we proposed to assess the response of CD4+ and CD8+ T lymphocytes from women who were infected by ZIKV during pregnancy 2–3 years ago. For this, two groups of women were compared according to the clinical outcome of their children: women who had asymptomatic children and women who had children born with CZS. The cells were stimulated in vitro with ZIKV peptides directed to T cells. After stimulation, IFN-γ production and cytotoxic activity (according to CD107a expression) were evaluated. Since chemokine receptors have been reported to be important molecules in the antiviral immune response [14,15,16], we evaluated the expression of CCR5 and CX3CR1 receptors and correlated this with donor T cell functionality.

In the present study, we proposed to evaluate the inflammatory and cytotoxic profiles of CD4+ and CD8+ T lymphocytes of ZIKV-infected women during pregnancy 2–3 years ago, and to correlate the functionality of T cells with the expression of CCR5 and CX3CR1 receptors.

## 2. Materials and Methods

### 2.1. Study Design, Volunteers, and Samples

A cross-sectional study was carried out in the 22 women infected with ZIKV who reported rash, with or without other clinical symptoms suggestive of arboviruses infections, such as arthralgia, myalgia, fever, headache, and conjunctival hyperemia during pregnancy overlapping with the ZIKV Public Health Emergency of National Concern in Brazil period (November 2015 and May 2017). This donors’ cohort was referred from the Exanthematic Diseases Unit at the Hospital Universitário Antonio Pedro of the Universidade Federal Fluminense (HUAP/UFF) located in Niteroi, Rio de Janeiro (Brazil). Laboratory evidence for ZIKV infection during pregnancy was based on mother’s positive RT-qPCR test results on serum and/or urine samples, which were done at the Central laboratory of Rio de Janeiro State (LACEN, RJ, Brazil) [17]. Exclusion criteria was mothers who were positive with a RT-qPCR for other arboviruses such as Chikungunya and Dengue. Moreover, mothers who presented positive test results to *Treponema pallidum* (syphilis), *Toxoplasma gondii* (toxoplasmosis), rubella, cytomegalovirus, and HIV infection were excluded. From 2018 to 2019, blood donations from these donors were collected at HUAP/UFF to be used in our study. Previous exposure to DENV or ZIKV were determined by the presence of detectable DENV-specific IgG titers [18] or ZIKV-specific IgG titers [19].

The control groups were comprised of 5 healthy donors who were never exposed to DENV or ZIKV (DENV-/ZIKV-) and 5 healthy donors who were never exposed to ZIKV but were exposed to DENV (DENV+/ZIKV-). All were similar in age compared to the group of mothers. It is important to bear in mind that over the last 30 years, extensive dengue epidemics have occurred in Brazil, which were characterized by the emergence and reemergence of different serotypes, changes to the epidemiological profile, and increasing numbers of serious and fatal cases [20]. Thus, recruiting Brazilians who have never been exposed to DENV in their lives is a difficult task but not an impossible one.

### 2.2. Detection of Dengue IgG and Zika Antibodies with an In-House Enzyme-Linked Immunosorbent Assay (ELISA)

Serum samples were tested using ELISA IgG specific for ZIKV and DENV. Detection of dengue-specific IgG antibodies (Panbio, Brisbane, Australia) and Zika-specific IgG antibodies (Euroimmun, Lübeck, Germany) were performed according to the manufacturer’s protocol. These ELISA were standardized to be used as recommended after studies by the Brazilian Ministry of Health.

### 2.3. The Plaque Reduction Neutralization Test (PRNT)

The PRNT was done for the laboratory verification of Zika cases. The cutoff value for PRNT positivity was defined as 90% (PRNT_90_). The ZIKV/H.sapiens/Brasil/ES2916/2015 strain identified in the State of Espírito Santo, Brazil was used. Samples with neutralizing antibodies for ZIKV were also submitted to PRNT_90_ for dengue virus serotype 1 (DENV-1 from West Pacific). The PRNT_90_ was realized to determine the maximum plasma dilution (1:10 to 1:320) needed to reduce arbovirus plaque formation by 90% among Vero CCL-81 cells, following standard protocols [21,22]. Before neutralization testing, plasma samples were heat-inactivated (56 °C, 30 min). Then, a final volume of each inactivated plasma samples and virus mixture was transferred to a well containing Vero cells and then initially screened at a dilution of 1:10 in 6-well plates at 37 °C for 60 min. Those that neutralized ZIKV by at least 90% were further tested at serial two-fold dilutions to determine 90% endpoint titers. Plasma samples were considered having DENV-neutralizing antibodies when a plasma dilution of at least ≥10 reduced no less than 90% of the formation of DENV viral plaques.

### 2.4. PBMC Isolation

Briefly, peripheral blood mononuclear cells (PBMCs) and plasma were isolated by Ficoll-PaqueTM PLUS density gradient centrifugation (GE Healthcare, Chicago, IL, USA) and frozen in fetal bovine serum (FBS, Gibco, Invitrogen Co., Waltham, MA, USA) containing 10% (vol/vol) dimethyl sulfoxide (Sigma-Aldrich, St. Louis, MO, USA). Cells were thawed on the day of the experiment and were used directly for in vitro assay.

### 2.5. ZIKV MegaPool (MP) Description

The ZIKV peptide megapool was designed and validated, as previously reported [13]. Briefly, a consensus sequence was derived from data retrieved from 103 sequences of ZIKV polyprotein proteome from different sites that had an outbreak of the disease (Canada, Africa, Asia, and the Americas), 42 of which were from the Americas [23]. Then, the consensus sequence was mapped to the ZIKV BeH818995 isolate and overlapping 15-mer by 10 amino acid residues peptides were synthesized as crude material (A&A, San Diego, CA, USA). The corresponding peptides were resuspended in DMSO, pooled, and then followed a sequential lyophilization to generate the MegaPool (MP) [24]. The length of the peptides contained in this MegaPool and the fact that no prediction filtering was applied allows the parallel identification of both ZIKV-specific CD4 and CD8 T cell reactivity.

### 2.6. IFN-γ ELISPOT Assay

Frozen collected PBMCs were assayed for IFN-γ cell responses, as previously described [13]. Briefly, mouse anti-human IFN-γ antibody (clone 1-DK1; Mabtech, Stockholm, Sweden) was added to 96-well plates (Multiscreen HTS; Millipore, Burlington, MA, USA) coated at 2.5 μg/mL, diluted in Phosphate Buffer Saline (PBS), pH 7.2-7.4 (Sigma-Aldrich, St. Louis, MO, USA). Then, PBMCs were added in triplicate to wells (2 × 10^5^ cells/well) in the presence or not of ZIKV MPs at 1 µg/mL, which was followed by incubation at 37 °C, 5% CO_2_ for 18 to 20 h. After washing with PBS–Tween 20, 1 μg/mL of IgG biotinylated anti-human IFN-γ (clone 7-B6-1; Mabtech) was added and incubated for 2 h at room temperature. After washing, streptavidin–alkaline phosphatase substrate (Mabtech) was prepared and added to the plate for 1 h at room temperature. Plates were washed, and alkaline–phosphatase substrate 5-bromo-4-chloro-3-indolyl-phosphate/nitro blue tetrazolium chloride (BCIP-NBT); (Kirkegaard & Perry Lab Inc., Gaithersburg, MD, USA) was added, allowing spots to develop. The reaction was stopped by washing with tap water. Spots were counted using an automated ELISPOT reader (ImmunoSpot1S6UV Ultra, Cleveland, OH, USA). The number of IFN-γ-producing cells was expressed as spot-forming cells (SFC) relative to 10^6^ PBMCs. Values were calculated by subtracting the number of spots detected in unstimulated control wells. Values were considered positive if they were equal or greater than 20 spots and at least two times above the mean of unstimulated control wells. The stimulation with phytohemagglutinin (PHA 5 µg/mL) will be done for all individuals analyzed as a positive control of in vitro stimulation.

### 2.7. Intracellular Cytokine Staining

PBMCs (2 × 10^5^ cells/well) were cultured 6 h with 1 μg/mL ZIKV MPs and brefeldin A [13]. Subsequently, the stimulated PBMC were stained with the Abs used for flow cytometry experiments listed in Supplementary Table S1. Then, the intracellular cytokine staining (ICS) was performed, after which it was permeabilized with saponin (0.05%) and stained with anti-IFN-γ antibody. The stimulation with phorbol and ionomycin myristate acetate (PMA/Ionomycin) was performed for all individuals analyzed as a positive control of in vitro stimulation. The data were collected using a BD FACS ARIA IIu flow cytometer and analyzed using FlowJo 10.5.2 software (Tree Star, Ashland, OR, USA).

### 2.8. Statistics Analysis

The statistical analyses were performed using the Kruskal–Wallis test or Friedman’s test followed by Dunn’s multiple comparisons test in each donor group. In addition, it was performed the Wilcoxon matched-pairs signed rank test between unstimulated (-) and ZIKV MPs stimulation in each group of mothers. Asterisks indicate significant differences (* *p* < 0.05, ** *p* < 0.01, *** *p* < 0.001, **** *p* < 0.0001) were considered significant using GraphPad PRISM (version 5) (GraphPad Software).

### 2.9. Study Approval

This approved study is titled Clinical follow-up of pregnant women with rash and their children: prospective study cohort” with approval number 56913416.9.0000.5243 on 29 March 2017. 

## 3. Results

### 3.1. The Serological Status by Detection of ZIKV and DENV-Specific IgG Antibodies and by Neutralizing Antibodies to ZIKV and DENV in Donors with a History of ZIKV Infection

ZIKV and DENV-specific IgG antibody detection was determined using the commercial capture ELISA assay. Among symptomatic mothers who were tested RT-qPCR +, 13 out of 22 (59.1%) presented both anti-ZIKV IgG and anti-DENV IgG. Similar percentages were observed between mothers who had asymptomatic (seven out of 12; 58.3%) and those whose children had CZS (six out of 10; 60%) children. Five out of 22 (22.7%) had anti-ZIKV IgG but not anti-DENV IgG, and these comprised four whose children were asymptomatic and one whose child had CZS children. Only one mother who had a child with CZS had anti-DENV IgG but not anti-ZIKV IgG. Another mother who had an asymptomatic child and two whose children had CZS had neither anti-DENV IgG nor anti-ZIKV IgG. To confirm previous ZIKV and DENV exposure, plasma samples were tested by means of PRNT90 for the detection of ZIKV and DENV-1-neutralizing antibodies. We could not perform PRNT for all four DENV serotypes since the plasma volume was insufficient for this. Thus, we chose to evaluate the DENV serotype 1, since this was the one with highest prevalence in Rio de Janeiro in the period in which the samples were collected (2015–2016) [25]. Out of 18 mothers who had anti-ZIKV IgG, 17 (94.4%) presented ZIKV-neutralizing antibodies with PRNT_90_ titer >320, thus confirming exposure to ZIKV. Interestingly, out of 13 mothers who had both anti-ZIKV IgG and anti-DENV IgG that were detectable, 10 had PRNT_90_ tests positive for ZIKV and DENV, thus suggestive of exposure to both viruses (Table 1).

### 3.2. Cell Responses to ZIKV MP Peptides Targeting T Cells, Among ZIKV and/or DENV-Immune Donors

**Figure 1:**
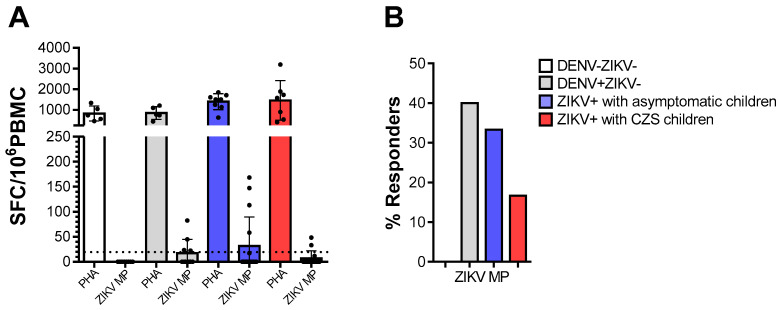
Ex vivo reactivity to Zika virus (ZIKV) peptides in ZIKV and/or dengue virus (DENV)-immune donors. The cell response was measured through IFN-γ production in ex vivo experiments using the ELISPOT as above, on cells from DENV-ZIKV- (white, *n* = 5), DENV+ZIKV- (gray, *n* = 5) and mothers who had asymptomatic children (blue, *n* = 9) and among whom had children with congenital Zika syndrome (CZS) (red, *n* = 7). (**A**) The magnitude of responses was expressed as the number of IFN-γ-secreting cells (SFC) per 10^6^ peripheral blood mononuclear cell (PBMC). Data are expressed as averages ± standard deviations. The statistical analyses were performed using the Kruskal–Wallis test followed by Dunn’s multiple comparisons test in each donor group. (**B**) The responses were considered positive if the net ratio of SFC/10^6^ PBMC constituted a stimulation index of ≥2. The statistical significance of differences in the frequency of response was determined using Fisher’s test. *p* values < 0.05 were considered significant.

We tested PBMC from donors who were DENV-/ZIKV-, DENV+/ZIKV-, or mothers who were Zika-positive (ZIKV+) during pregnancy, for reactivity against ZIKV peptides, in ex vivo IFN-γ ELISPOT assays. The vigorous responses of all individuals to mitogenic stimulation with PHA indicated that their cells were viable and able to respond to stimulation. As expected, cells from DENV-/ZIKV- donors did not respond to the ZIKV peptides. Not surprisingly, 40% of cells from DENV+/ZIKV- donors recognized ZIKV- peptides. Among the mothers who were ZIKV-positive during pregnancy, cells from 33.3% whose babies were asymptomatic babies and 16.7% whose babies presented CZS responded to ZIKV- peptides. Meanwhile, no statistical differences were found among the mothers who were ZIKV-positive during pregnancy (Figure 1).

### 3.3. CD4 and CD8 T Cell Responses to ZIKV MP Peptides from Women Who Had ZIKV Infection During Pregnancy

Then, we compared ZIKV T cell reactivity only in convalescent mothers infected with ZIKV during pregnancy. The child’s clinical outcome (i.e., born with or without CZS) was taken to be the variable. The ICS assays enabled evaluation of CD4 and CD8 responses and measurement of IFN-γ production. Together, expression of the CD107a molecule was evaluated as a function of the degranulation capacity of T cells.

**Figure 2:**
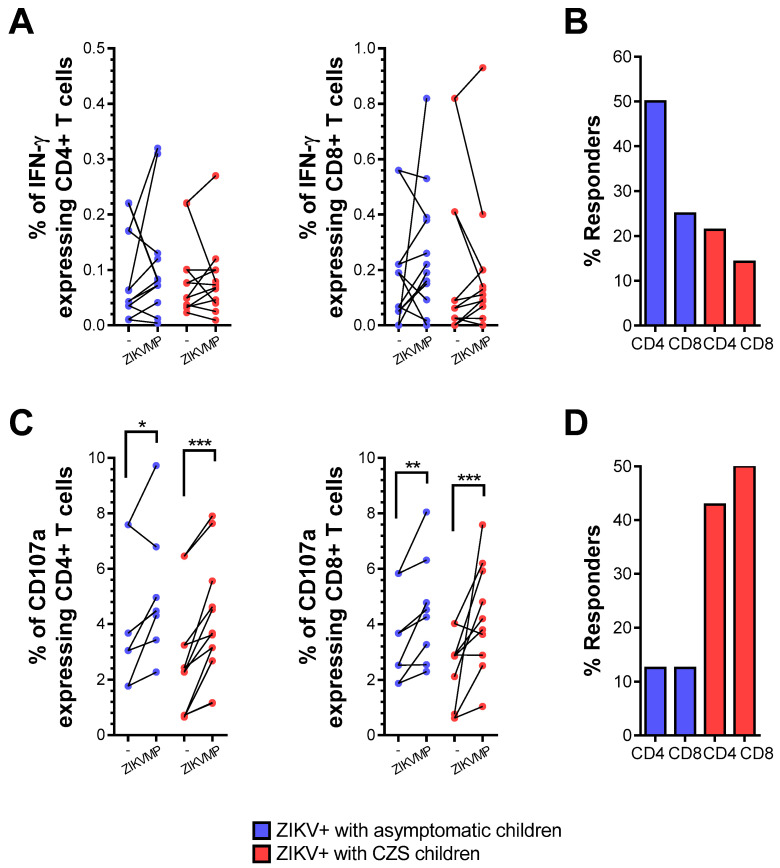
IFN-γ production and CD107a expression in T cells from mothers with a history of ZIKV infection. The ICS profiles of IFN-γ staining and CD107a expression in CD4 and CD8 T cells after stimulation with ZIKV MegaPool (MP) peptides are shown in a mother infected with ZIKV during pregnancy. Cells from mothers who had asymptomatic children (blue, *n* = 8–12) and among whom had children with CZS (red, *n* = 10). (**A**) Data are expressed as symbols and lines without ZIKV peptide stimulation (-) and after ZIKV peptide stimulation (ZIKV MPs), regarding the percentage of CD4+ and CD8+IFN-γ + cells. Statistical analyses were performed using the Wilcoxon matched-pairs signed-rank test between unstimulated (-) and ZIKV MPs stimulation in each group of mothers. (**B**) Responses were considered positive if the percentage of IFN-γ + cells with ZIKV MPs showed a stimulation index of ≥ 2 compared with the percentage of IFN-γ + cells without ZIKV MPs (-). Statistical significance for differences in frequency of responses was determined using Fisher’s test. (**C**) Data were shown in the same way as in (**D**), regarding the percentage of CD4+ and CD8+CD107a+ cells. Asterisks indicate significant differences (* *p* < 0.05, ** *p* < 0.01, *** *p* < 0.001).

The frequency of ex vivo responses in cells from mothers who had asymptomatic children was 50 and 25% for IFN-γ-producing CD4 and CD8 T cell responses, respectively. Marginal responses of IFN-γ-producing CD4 and CD8 T cells to ZIKV peptides were observed in relation to 21.4 and 14.3% of the cases of the mothers who had children with CZS (Figure 2A,B). Thus, after ZIKV convalescence, the responses of CD4 and CD8 T cells to ZIKV-restricted peptides were still higher in mothers who had asymptomatic children than in those who had children with CZS but were not significantly different in terms of either the magnitude response or the frequency of response.

However, during the convalescence phase, the mothers’ T cells showed appreciable reactivity in terms of increased magnitude of expression of CD107a in relation to ZIKV peptides (Figure 2C,D). These findings confirm that CD4 and CD8 T cells showed a degree of cytotoxic activity after ZIKV convalescence, which did not significantly differ according to the baby’s clinical outcome.

### 3.4. Expression of CCR5 and/or CX3CR1 Chemokine Receptors Relating to Functionality of CD4 T-Cells

Then, we evaluated the frequency of T cells expressing two chemokine receptors: CCR5 and/or CX3CR1. These chemokine receptors distinguish subpopulations of T cells and influence the migration profile and functionality of immune cells. The flow cytometry surface staining assay enabled these evaluations. We compared CD4+ T cells from convalescent mothers infected with ZIKV during pregnancy, taking the child’s clinical outcome as the variable.

**Figure 3:**
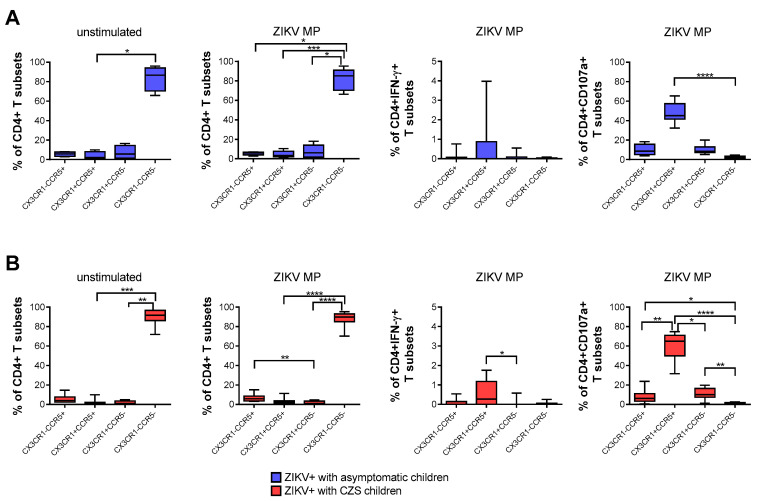
Expression of CCR5 and/or CX3CR1 correlated with the functionality of CD4 T cells from mothers with a history of ZIKV infection. Frequency of CD4+ T cells according to the expression of CCR5 and/or CX3CR1 in mothers who had asymptomatic babies (blue, *n* = 8–12) (**A**) and in those who had CZS children (red, *n* = 10) (**B**). Frequency observed after stimulation with ZIKV peptides in each group of mothers. The gates of IFN-γ-producing CD4+ T cells and CD4+CD107a+ were selected as an analysis strategy. Data are expressed as box and whiskers (min to max). Statistical analyses were performed using Friedman’s test followed by Dunn’s multiple comparisons test between the groups of mothers. Asterisks indicate significant differences (* *p* < 0.05, ** *p* < 0.01, *** *p* < 0.001, **** *p* < 0.0001).

A higher frequency of CD4+ T cells that expressed neither CCR5 nor CX3CR1 was found naturally and after stimulation with ZIKV MP peptides in both groups of mothers. After stimulation with in vitro ZIKV MP peptides, we selected the gate of IFN-γ-producing CD4+ T cells as the analysis strategy. It was observed that CD4 T cells that co-expressed CCR5+ and CX3CR1+ were the main producers of IFN-γ. A similar analysis was performed by selecting the gate of CD4+ CD107a+ T cells, which also demonstrated that cells with a cytotoxic profile co-expressed CCR5+ and CX3CR1+. These profiles were more clearly observed in mothers who had CZS children than in those with asymptomatic children (Figure 3A,B).

### 3.5. Expression of CCR5 and/or CX3CR1 Chemokine Receptors Correlated with the Functionality of CD8 T-Cells

**Figure 4:**
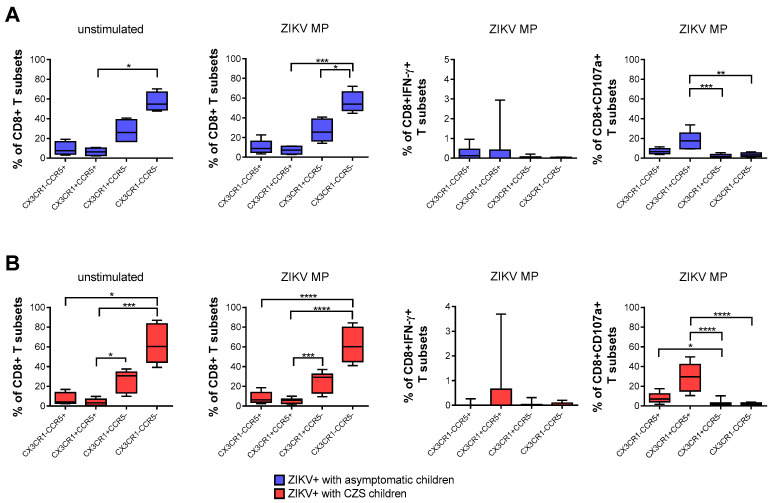
Expression of CCR5 and/or CX3CR1 correlated with the functionality of CD8 T cells of mothers with a history of ZIKV infection. Frequency of CD8+ T cells according to the expression of CCR5 and/or CX3CR1 in mothers who had asymptomatic babies (blue, *n* = 8–12) (**A**) and in those who had CZS children (red, *n* = 10) (**B**). Frequency observed after stimulation with ZIKV MP peptides in each group of mothers. The gates of IFN-γ-producing CD8+ T cells and CD8+CD107a+ were selected as an analysis strategy. Data are expressed as box and whiskers (min to max). Statistical analyses were performed using Friedman’s test followed by Dunn’s multiple comparisons test between the groups of mothers. Asterisks indicate significant differences (* *p* < 0.05, ** *p* < 0.01, *** *p* < 0.001, **** *p* < 0.0001).

A higher frequency of CD8+ T cells that express neither CCR5 nor CX3CR1, followed by CX3CR1+CCR5^neg^ CD8 T cells, was found naturally and after stimulation with ZIKV peptides in both groups of mothers. After stimulation with in vitro ZIKV peptides, we selected the gate of IFN-γ-producing CD8+ T cells. None of the subpopulations was the main producer of IFN-γ. By selecting the gate of CD8+ CD107a+ T cells, mothers became infected with ZIKV during pregnancy, which also demonstrated that cells with a cytotoxic profile co-expressed CCR5+ CX3CR1+, followed by CX3CR1^neg^CCR5+ CD8 T cells. These profiles were more clearly observed in mothers who had CZS children than in those with asymptomatic children (Figure 4A,B).

## 4. Discussion

Pregnancy has been described as a state of “immune suppression”, but contrary to this “myth”, the immune system in pregnancy is characterized by a reinforced network of cellular recognition, communication, traffic, and repair that enables the maintenance of well-being for the mother and fetus. On the other hand, the fetus represents a “singularity of the immune system” that changes the way in which the mother responds to the environment. Therefore, it is appropriate to refer to pregnancy as a unique, active, functional, and carefully modulated, but not suppressed, immunological condition [26]. Viruses gain access to the cells of the decidua and placenta by going up through the lower reproductive tract or via hematogenous transmission. Viral tropism for the decidua and/or placenta is dependent on the expression of the viral recognition receptor in the cells and on the maternal immune response. Although viral infections are common in pregnancy, transplacental passage and fetal infection are an exception [27].

The recent ZIKV outbreak in Brazil redirected attention to the risks associated with viral infections during pregnancy due to the surprising increase in the incidence of abnormalities in the central nervous system of fetuses [28,29,30]. Between November 2015 and October 2019, the Brazilian Ministry of Health reported 18,282 cases of changes in fetal growth and development, of which 3474 (19%) were related to ZIKV infection [31]. To search for correlations between ZIKV-induced alteration of maternal immunity and fetal abnormalities, Foo et al. (2018) performed extensive multiplexing analysis on cytokines. These authors revealed that the chemokines CXCL10, CCL2, and CCL8 were specifically associated with symptomatic ZIKV infection during pregnancy, and distinct immunoprofiles were detected in different trimesters in ZIKV-infected pregnant women. Intriguingly, high CCL2 levels and their inverse correlation with CD163, TNFRSF1A, and CCL22 levels was apparently associated with ZIKV-induced abnormal birth [32].

In an attempt to understand the priming of the immune response in relation to ZIKV infection during pregnancy, we evaluated the inflammatory and cytotoxic profiles of CD4+ and CD8+ lymphocytes specific to ZIKV among women who had become infected during pregnancy, and we correlated the functionality of T cells with the expression of CCR5 and CX3CR1. The response of T lymphocytes to ZIKV peptides was evaluated in 22 women, 2–3 years after they had had acute infection, regrouped according to the clinical outcome of their living children.

All the women included in this study had had a clinical and laboratory diagnosis of Zika, which was confirmed in the acute phase of the disease. As expected, in 63.6% of these women, anti-DENV IgG was detected, and 81.2% of them had anti-ZIKV IgG. Most of the commercial serological kits available on the market tend to have high sensitivity but low specificity due to the high cross-reactivity between arboviruses [33,34]. Only neutralization assays measure the biological parameters of virus neutralization in vitro; therefore, these are the most specific serological tests [35].

Based on PRNT_90_ data, 59% of the mothers who had neutralizing antibodies for ZIKV had already been exposed to DENV. Thus, we compared 13 mothers who were ZIKV+/DENV+ with 8 mothers who were ZIKV+/DENV-, but unfortunately, we did not find any significant difference, which was probably because of the small sample size for the comparison. It is not yet clear whether immunity prior to DENV cross-protects ZIKV infection during pregnancy. Contradictory datasets regarding the impact of antibody-dependent enhancement (ADE) on congenital malformations in babies in experimental animal’ models and human donors [36]. Regarding the mothers’ ZIKV T-cell effector immune response repertoires, the authors observed a reduction in a load of ZIKV in maternal and fetal tissues and an increase in fetal viability and growth in immune mice to DENV compared to non-immune mice. Interestingly, the depletion or genetic deficiency of CD8 T cells nullified this effect, even under ADE conditions [10]. Ninety-four Brazilian pregnant women who gave birth to infants with or without microcephaly during the ZIKV epidemic were evaluated. Based on this study, DENV immunity is not a prerequisite for ZIKV entry into the fetal compartment [37]. However, an increased replication of ZIKV-infected human placental tissues was seen in the presence of DENV antibodies [11]. Reynolds et al. have recently developed a study on pregnancy and immunity to ZIKV in human patients. Almost all mothers with a history of ZIKV infection have serological evidence of immunity to dengue. Furthermore, the authors showed that multifunctional responses were induced to E and NS1 ZIKV antigens, while immunoregulatory responses were also induced to NS5 and C ZIKV proteins, which could impact the clinical outcome’s children [38].

Farther than the context of pregnancy or congenital infection, several studies have been evaluating the T cell responses to ZIKV proteins in human donors’ dengue immune. Indeed, ZIKV-reactive T cells in the acute phase of infection are detected earlier and in greater magnitude in DENV-immune individuals. The pattern of CD4 responses to ZIKV-restricted class II peptides was remarkably similar in acute and convalescent phases, while a lower frequency and magnitude of responses were observed with the CD8 counterpart [13]. In contrast, an immunological signature for CD8 T cell responses was temporally stable after Ag-specific stimulation even 1–2 years after ZIKV infection [39]. Moreover, circulating tetramer-positive ZIKV-specific CD8 T-cells peaked at early convalescence day post-infection with elevated levels persisting for months from a volunteer woman [40]. Instead, whereas C-, prM-, E-, and NS5-specific cytokine-expressing CD4 T cells were readily detected in all patients tested, absent or low detection of functional CD8 effector T cells against peptides spanning ZIKV C, prM, E, and NS5 were identified [41].

An increase in the expression of CCR5 in T cells and a decrease in CCL5 plasma levels, in patients in the acute phase of dengue, have already been demonstrated by our group. In addition, we found a high frequency of CCL5+ cells in the liver tissue of fatal dengue cases. The presence of these cells in liver tissue might be involved in the establishment of an intense tissue inflammatory process and, consequently, in the activation of the tissue antiviral immune response [42]. Mice infected with DENV and treated with Met-RANTES, a potent CCR5 inhibitor, were found to have lower viral load and increased survival, thus indicating that inhibition of the CCL5/RANTES interaction through CCR5 may be an important therapeutic target in dengue [43]. Regarding CX3CR1, Weiskopf et al. (2015) demonstrated that CD4+ T lymphocytes specific to DENV express CX3CR1 and that these cells are characterized by their cytolytic activity. These cytolytic CD4+ lymphocytes are more frequent in DENV immune-donors that carry a protective HLA-DR allele, which would lead to greater control of viral infection in vivo [44]. We studied the responses of T cells and the expression of chemokine receptors in donors infected by ZIKV or DENV, or coinfected by DENV and ZIKV. We observed increases in the expression of CCR5, CX3CR1, and CXCR3 in CD4+ and CD8+ T cells among patients, thus indicating that these cells have an activation profile and/or migratory capacity that is independent of the virus [45].

We initially demonstrated the immunocompetence of the donors in our study according to ELISPOT and ICS data, using polyclonal stimuli. We also emphasize that we had previous knowledge that the ZIKV peptides used in this study were immunogenic, as demonstrated in the study by Grifoni et al. (2017) [13].

After in vitro stimulation with ZIKV peptides with IFN-γ production, cells from mother’s immune to ZIKV who had asymptomatic children showed a tendency toward greater magnitude of response and a tendency toward a higher frequency of response compared with mothers who had children with CZS. Interestingly, the CD4+ T cell responses tended to be greater than the CD8+ T cell responses in both groups of mothers.

A similar analysis was performed regarding the expression of CD107a. Conversely, regarding the inflammatory response with IFN-γ production after in vitro stimulation with ZIKV peptides, the cells of mothers who had asymptomatic children showed a tendency toward lower frequency of response, compared with those of mothers who had children with CZS. The frequencies of response of CD4+ and CD8+ T cells were quite similar in the two groups of mothers.

In the light of these preliminary data, we suggest that in cases of acute ZIKV infection during pregnancy, there could be differences in the priming of the mothers’ T cells. The T cells of mothers who had asymptomatic children would have tended toward a more inflammatory profile, while the T cells of mothers who had children with CZS tended toward a more cytotoxic profile. This priming was demonstrated 2–3 years after infection through in vitro stimulation tests with ZIKV peptides. It remains to be seen whether this differential T-cell response would, in fact, have influenced the clinical outcome of babies exposed to ZIKV during pregnancy.

A previous study demonstrated that in dengue hemorrhagic fever, most activated T cells had an inflammatory profile, with production especially of IFN-γ and TNF-α, but they were less cytotoxic, with a low expression of CD107a. However, in patients with a better outcome, such as dengue fever, this profile was shown to be reversed; most T cells had a more cytotoxic and less inflammatory profile. In summary, a worse prognosis for dengue would be involved with an inflammatory response, while there would be a better prognosis with a cytotoxic response [46].

It is difficult to extrapolate data from the literature that were based on acute infection by DENV or ZIKV with the data from our study. Here, all the mothers were symptomatic, with a similar clinical course in the acute phase of Zika. The difference between them was in their children’s clinical outcome. Barros et al. (2018) showed that differential levels of cytokines and chemokines were present during acute ZIKV infection. These related to specific clinical symptoms, which suggested that they participated in underlying mechanisms and might also participate in controlling the virus. Those authors highlighted the mediators CXCL10 (protein 10 induced by IFN-γ), CCL5, IFN-γ, IL-9, IL-7, IL-5, and IL-1ra, which are predominantly present in acute ZIKV-infected individuals, rather than in healthy donors [47].

An experimental approach closes to ours was used by Reynolds et al. (2020). These authors evaluated Brazilian women who gave birth to infants with microcephaly following ZIKV exposure in the 2015-16 outbreak. Similar to the women in our study, most of them had dengue immunity. Their responses to Env and NS1 ZIKV peptides were polyfunctional, with cells producing IFN-γ, TNF-α, IL-4, IL-13, and IL-10. In contrast, their responses to NS5 ZIKV peptides only produced the immune regulatory TGF-β1 cytokine. Thus, these authors’ data support an argument that different viral products may skew the antiviral response to a more pro or anti-inflammatory outcome, with an associated impact on immunopathogenesis [48].

Whether the T cells of mothers who had asymptomatic children would have been primed for a more inflammatory profile, while the T cells of mothers who had children with CZS would have been primed for a more cytotoxic profile, and whether that would have influenced the clinical outcome of babies exposed to ZIKV during pregnancy, needs further investigation. This could even use other peptides from ZIKV to confirm this differential response profile.

During an inflammatory process, there is an increase in the secretion of chemokines, in addition to an increase in the expression of their respective receptors in target cells. This occurs to control and coordinate the migration of immune cells from the infection site to secondary lymphoid organs and to sites of inflammation, to initiate an effective immune response [49].

In this study, we extrapolated our analyses through observing the magnitude of IFN-γ production and CD107a expression in relation to the expression of CCR5 and CX3CR1 after in vitro stimulation with ZIKV peptides. Here, higher frequencies of CD4+ and CD8+ T cells that expressed neither CX3CR1 nor CCR5 in the two groups of mothers were observed. Even after in vitro stimulation with ZIKV peptides, these frequencies remained unchanged. However, in stimulation with ZIKV peptides, T cells co-expressing CX3CR1+ CCR5+ were the main producers of IFN-γ, and this was much clearer in the CD4+ T cells of mothers who had children with CZS. Similarly, the highest frequency of T cells expressing CD107a was CX3CR1+ CCR5+, which was also clearly seen in mothers who had children with CZS. In this group of mothers with CZS children, an important proportion of CD8+ T cells that expressed CX3CR1^neg^CCR5+ co-expressed CD107a.

Böttcher et al. showed that CX3CR1 was a marker of memory CD8+ T cells with cytotoxic function, which will help to deepen the understanding of the principles of T cell memory and immune protection. In their study, they detected virus-specific CX3CR1+ GzmB+ CD8+ T cells in patients with chronic viral infections, which suggested that continuous antiviral immunity was present, but without resolution of the infection [50]. Likewise, dengue hemorrhagic fever patients have been found to have worse evolution, in which most T cells had a more inflammatory and less cytotoxic profile [46]. Here, we found a higher frequency of CD107a+ CD4+ and CD8+ cytotoxic T cells co-expressing CX3CR1+ CCR5+, which was much clearer in the T cells of mothers who had children with CZS. Thus, it can be asked whether it is possible that this more cytotoxic profile in Zika is associated with a worse result. These data are still preliminary, but we intend to continue the analyses to confirm the functionality of these cells and, in addition, to use other peptides derived from ZIKV.

Recently, a study evaluated a library of 671 synthetic peptides covering the whole polyprotein of ZIKV to stimulate PBMCs in individuals 2 years after exposure to ZIKV. Interestingly, the authors of that study observed that the quality of ZIKV-specific T cell responses depended on the region of the ZIKV protein. Thus, they detected that IFN-γ and TNF-α production were especially stimulated by prM, capsid, or NS1 in CD8+ T cells and by capsid or prM in CD4+ T cells. In addition, there was an increase in the frequency of IL-10+ CD8+ T cells after stimulation with prM, capsid, NS1, NS3, or NS5. Multifunctional properties were observed in ZIKV-specific T cells responding especially to prM, capsid, NS1, or, to a smaller extent, NS3 antigens. In addition, CD8+ T cells were more prone to a multifunctional phenotype than were CD4+ T cells [51].

**Figure 5:**
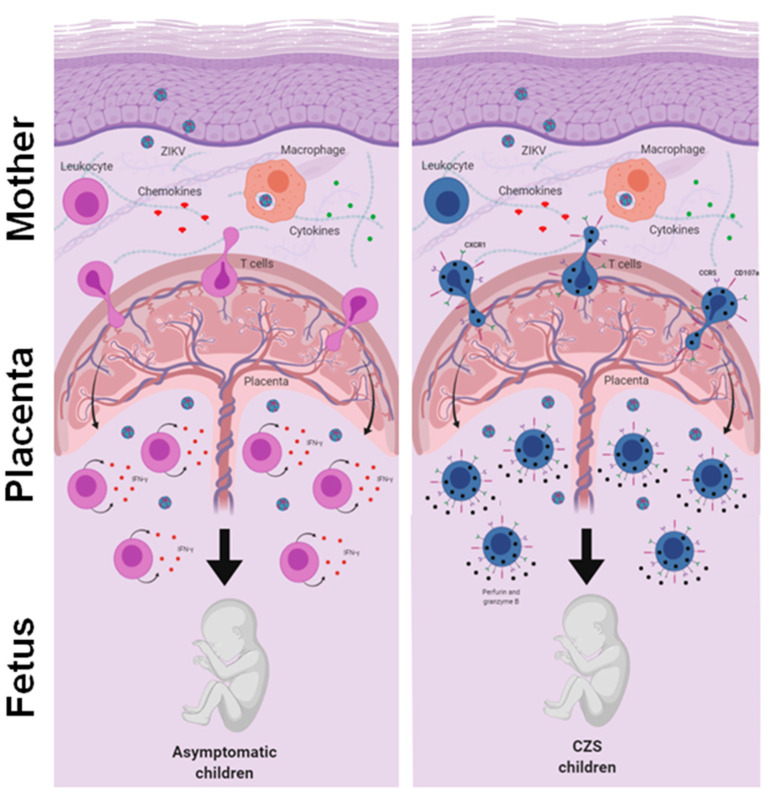
An integrative view of the fetal-placental anti-ZIKV immune response and the maternal immune system. The immune system in pregnancy is characterized by a reinforced network and repair to maintain the well-being of the mother and fetus. Although viral infections are common in pregnancy, transplacental passage and fetal infection are an exception. Here, we compare the reactivity of T cells to ZIKV peptides in recovery mothers infected with ZIKV during pregnancy. The child’s clinical outcome (that is, born with or without CZS) was considered the variable. We observed a trend toward high responses of IFN-γ producing T cells in mothers who had asymptomatic children and a higher expression of CD107a in T cells in mothers who had children with CZS. In addition, we found a higher frequency of CD107a+ expressing T cells and co-expressing CX3CR1+ CCR5+, which was much clearer in the T cells of mothers who had children with CZS. We suggest that this differential profile influenced the clinical outcome of babies, although these data need to be further investigated.

Thus, we evaluated the response of T cells to ZIKV peptides from women who were infected with ZIKV during pregnancy 2 to 3 years ago. Our data did not show any notable differences between the mothers who gave birth to children with CZS and those who gave birth to asymptomatic children. Although no study has yet addressed this question, it is possible that marked differences between the groups that occurred in the acute phase of Zika may not have been so evident in the recovery phase. Meanwhile, we observed a tendency toward a higher IFN-γ-producing T cell response in mothers who had asymptomatic children and a higher CD107a expression in T cells in mothers who had children with CZS. In addition, we found a higher frequency of CD4+ and CD8+ cytotoxic CD107a+ T cells co-expressing CX3CR1+ CCR5+, which was much clearer in the T cells of mothers who had children with CZS. Perhaps this differential profile would have influenced the clinical outcome of babies exposed to ZIKV during pregnancy. These data need to be further investigated, including the evaluation of other ZIKV peptides and other markers and functional assays (Figure 5).

## Figures and Tables

**Table 1:** Characteristics of the recovered mothers infected to Zika virus during pregnancy recruited from 2018 to 2019.

Group	Outcome at Birth	ID	Age ^a^	Illness Time ^b^	Gestational Trimester at Onset Rash	State	RT-aPCR ZIKV	ZIKV Anti-IgG	DENV Anti-IgG	PRNT 90 ZIKV	PRNT 90 DENV-1
Mothers	Asympt.	M1	23	29	1st	RJ	pos	pos	neg	≥320	≥10
	Asympt.	M2	29	19	2nd	RJ	pos	pos	pos	≥320	≥10
	Asympt.	M3	22	38	3rd	RJ	pos	pos	neg	160	<10
	Asympt.	M4	36	40	3rd	RJ	pos	pos	pos	≥320	≥10
	Asympt.	M5	41	x	2nd	RJ	pos	neg	neg	≥320	<10
	Asympt.	M6	21	38	2nd	RJ	pos	pos	pos	≥320	≥10
	Asympt.	M7	37	38	2nd	RJ	pos	pos	pos	≥320	≥10
	Asympt.	M8	23	24	2nd	RJ	pos	pos	neg	≥320	<10
	Asympt.	M9	33	38	2nd	RJ	pos	pos	pos	<10	<10
	Asympt.	M10	27	40	2nd	RJ	pos	pos	neg	≥320	≥10
	Asympt.	M11	30	39	2nd	RJ	pos	pos	pos	≥320	≥10
	Asympt.	M12	37	38	2nd	RJ	pos	pos	pos	≥320	≥10
			29.5 (21–41)	38 (19–40)							
Mothers	CZS	M13	24	36	2nd	RJ	pos	neg	neg	≥320	<10
	CZS	M14	40	40	2nd	RJ	pos	pos	pos	≥320	≥10
	CZS	M15	42	39	2nd	RJ	pos	pos	pos	≥320	<10
	CZS	M16	23	23	1st	RJ	pos	neg	neg	160	<10
	CZS	M17	21	42	1st	RJ	pos	pos	pos	≥320	≥10
	CZS	M18	28	41	1st	RJ	pos	neg	pos	≥320	≥10
	CZS	M19	24	23	1st	RJ	pos	pos	neg	≥320	<10
	CZS	M20	33	39	2nd	RJ	pos	pos	pos	≥320	≥10
	CZS	M21	41	36	2nd	RJ	pos	pos	pos	≥320	<10
	CZS	M22	21	39	before	RJ	pos	pos	pos	≥320	<10
			26 (21–42)	39 (23–42)							

Age ^a^, years; Illness time ^b^, months; median (minimum–maximum).

## Data Availability

Data sharing not applicable. No new data were created or analyzed in this study. Data sharing is not applicable to this article.

## Supplementary Materials

The following are available online at https://www.mdpi.com/1999-4915/13/2/191/s1, Table S1: Antibodies used in BD FACS ARIA IIu flow cytometer.
