# Supplementary material for: Evaluation of the Expression of CCR5 and CX3CR1 Receptors and Correlation with the Functionality of T Cells in Women infected with ZIKV during Pregnancy
Source: Viruses. 2021 Jan 28;13(2):191. doi: 10.3390/v13020191 (PMC7912595; doi:10.3390/v13020191)
Supplement: Supplementary file 1 [file viruses-13-00191-s001.pdf]

Supplementary Table S1: Antibodies used in BD FACS ARIA IIu flow cytometer

**Target Molecule**	**Clone**	**Company**	**Catalog #**
IFN-γ	4S.B3	eBioscience	11-7319-82
CCR5	J418F1	Biolegend	359124
CD3	UCHT1	BD Bioscience	557943
CD4	SK3	BD	347327
CD8	3B5	Invitrogen	MHCD0817
CX3CR1	2A9-1	Biolegend	341612
CD107a	H4A3	Biolegend	328630

Supplementary Table S2: Plasma levels of immune mediators were evaluated in women who had asymptomatic children and those who had children with CZS.

Immune mediators	Outcome at birth		p
	Assympt.	CZS	
**MCP-1/CCL2**	60.9 (21.7 - 67.2)	61.1 (0.0 - 83.5)	>0.999
**MIP-1α/CCL3**	0.2 (0.1 - 0.5)	0.3 (0.1 - 0.8)	0.535
**MIP-1β/CCL4**	13.5 (4.1 - 15.4)	7.8 (5.0 - 15.0)	0.761
**RANTES/CCL5**	10.4 (5.5 - 19.0)	17.6 (8.0 - 33.1)	0.240
**Eotaxin/CCL11**	12.1 (5.0 - 22.9)	5.4 (2.8 - 31.0)	0.486
**CXCL10/IP-10**	24.5 (9.8 - 172.0)	57.8 (30.9 - 122.2)	0.209
**G-CSF**	26.0 (19.5 - 31.1)	25.7 (17.4 - 36.8)	0.859
**IFNγ**	91.1 (81.8 - 142.3)	97.6 (78.4 - 163.6)	0.649
**IL-1ra**	209.7 (162.9 - 236.9)	246.6 (134 - 337.1)	0.411
**IL-1β**	0.6 (0.0 - 0.08)	0.4 (0.1 - 0.6)	0.326
**IL-4**	0.5 (0.3 - 0.7)	0.4 (0.3 - 1.2)	0.889
**IL-5**	0.7 (0.5 - 1.0)	1.0 (0.6 - 1.9)	0.150
**IL-6**	2.8 (2.4 - 5.1)	3.0 (0.9 - 5.8)	0.702
**IL-7**	1.7 (1.4 - 2.8)	3.2 (1.3 - 4.8)	0.189
**IL-8**	14.1 (6.5 - 19.0)	22.4 (4.5 - 28.7)	0.432
**IL-9**	0.0 (0.0 - 8.5)	5.3 (0.0 - 17.8)	0.312
**IL-12p70**	4.6 (3.1 - 8.2)	2.8 (2.2 - 5.1)	0.179
**IL-13**	0.4 (0.2 - 0.7)	0.4 (0.2 - 1.1)	0.765
**IL-17A**	8.7 (6.3 - 12.3)	10.3 (6.8 - 14.6)	0.703
**PDGF-BB**	250.5 (174.2 - 445.6)	159.7 (118.6 - 419.4)	0.349
**TNFα**	9.3 (4.2 - 15.1)	9.3 (4.0 - 10.1)	0.704
**VEGF**	33.6 (23.8 - 50.1)	21.0 (15.2 - 28.1)	0.132

median (minimum – maximum)

Differences between groups were calculated using unpaired t test or Mann-Whitney test
